# Ladder Safety: A Taxonomy of Limb-Movement Patterns for Three Points of Control

**DOI:** 10.3390/ijerph17082897

**Published:** 2020-04-22

**Authors:** Roger C. Jensen, Charles J. Holland

**Affiliations:** Safety, Health and Industrial Hygiene Department, Montana Technological University, Butte, MT 59701, USA; cholland@mtech.edu

**Keywords:** ladder climbing, falls, occupational safety, construction, risk, training

## Abstract

Traditional guidance on ladder safety emphasizes training workers on the use of three points of contact. More recent guidance is to train workers to use three points of control. What is lacking is empirical information about what limb-movement patterns effectively support the use of three points of control. This project was conducted to establish a taxonomy of possible limb-movement patterns and a means for comparing relative safety. Prior to the experiment, a taxonomy of six possible limb-movement patterns was established. A sample of 20 undergraduate students performed four tasks each without any instructions on limb-movement pattern. The tasks were ascending and descending a straight ladder and a portable ladder, once each, while being videotaped. Out of 80 observed tasks, 59 of the subjects were using rungs rather than rails. Analysis of rung users identified the use of all six patterns. An innovative measure of safe performance was developed and used to compare the patterns. Statistical analysis did not find significant differences in the patterns based on the safety performance measure.

## 1. Introduction

Falls from ladders result in many occupational fatalities and injuries. The U.S. Bureau of Labor Statistics (BLS) reported a review of occupational fatality data for the United States [1]. In the six years studied (2011–2016), there were 3723 fatal falls to lower level. Out of these fatal falls, more than half occurred in the construction sector, and the two most common sources were falls from ladders (836) and falls from roofs (763) [1].

The annual average for the 6-year period was 139 fatal falls from ladders. This increased to 145 in 2018 [2]. The construction industries contributed 88 of the 145 fatal falls [2]. These grim records are among many that document the importance of finding better ways to prevent ladder falls.

### 1.1. Risk Reduction through Training

Instructing employees on ladder-climbing techniques is a recognized tactic for preventing ladder falls. A traditional instruction has been to use three points of contact when ascending and descending ladders. That requires climbers to have only one limb moving at a time, and it considers hand contact with either a rung or the siderail as being sufficient. Results of experimental studies of human gripping capabilities indicate significantly more gripping capability when using the rungs compared to using the side rails [3,4,5]. This is particularly critical during the brief time when one foot is moving because the other foot must support most of the climber’s weight [6,7]. If the rung-connected foot slips, a fall will start and the hands will need to have a firm grip to allow recovery. Gripping the rungs provides much greater holding power than gripping the siderails [3,4,5,6,7]. Thus, the new guideline for ascending or descending a ladder is to maintain three points of control, which means the hands must be using the rungs, not the siderails.

The literature found on ladder safety indicated broad recognition of the hazards of using ladders, especially in the construction industries. Regulations of the U.S. Occupational Safety and Health Administration [8], as well as general guidance [6], point to training as an important method for reducing the risk of falls involving ladders. A survey of construction-industry roofing subcontractors found strong preference for training by demonstration rather than by classroom or reading [9]. Therefore, if training on ladder safety is used, it makes sense to use demonstration as a major component of the training. To demonstrate how to safely ascend and descend a ladder, the instructor needs to know precisely what limb-movement patterns can achieve three points of control. This is more complex than simply giving trainees verbal instructions to use three points of control.

### 1.2. Safety in Ladder Climbing

We found reports of several studies addressing safety aspects of ascending and descending ladders. One line of research has been examining the response of climbers when a foot slips off a rung [10,11,12,13]. Another examined the required friction to avoid a foot slipping from a rung on ladders set at three different angles [14].

A number of researchers have addressed the matter of limb-movement patterns. These descriptions typically define a stride as the movement of all four limbs in some order, starting with a foot movement. Pliner and Beschorner [11] describe four movement patterns:

Two-beat lateral;
Two-beat diagonal;
Four-beat lateral;
Four-beat diagonal.

The two-beat lateral pattern involves nearly simultaneous movement of the right hand and foot alternating with the left hand and foot. The two-beat diagonal pattern involves nearly simultaneous movement of the right foot and left hand, alternating with movement of the left foot and right hand. Because both two-beat patterns necessarily involve the simultaneous air time of two limbs, neither will achieve the desired three points of control. Both four-beat patterns involve moving individual limbs in order, therefore, these patterns have potential to support the desired three points of control.

Some earlier studies of ladder-climbing biomechanics established a foundation for more recent studies. Dewar pioneered ladder-climbing research with a laboratory study of ladder ascending patterns using the two-beat diagonal pattern [15]. Bloswick and Chaffin studied the biomechanics of ascending a straight vertical ladder using a two-beat lateral climbing pattern [16].

Studies in which subjects were not instructed to use particular climbing patterns have found that preferred climbing patterns depend on ladder angle [11,17,18]. For a portable ladder leaning at a 75° angle, McIntyre reported the pattern-specific percentages of his 22 male subjects [17]. They used the two-beat lateral the most (30.3%), followed by the four-beat lateral (28.8%), the two-beat diagonal (18.2%), and the four-beat diagonal (15.2%). For a straight vertical ladder, Pliner and Beschorner reported the climbing patterns their 38 subjects were using just before a rung perturbation occurred [11]. The patterns used by their subjects were, in order, the four-beat diagonal (31.3%), the two-beat lateral (28.2%), the two-beat diagonal (22.5%), and the four-beat lateral (17.5%).

Hammer and Schmalz studied effects of ladder inclination on limb-movement pattern using 28 subjects [18]. They found pattern preferences depended on whether ascending or descending as well as the ladder angle. For example, for ascending they found:

At 90°, two-beat lateral was preferred;
At 70°, four-beat lateral was preferred;
At 50°, four-beat diagonal was preferred.

No studies were found that provide a science-based justification for teaching particular limb-movement patterns for ascending and descending ladders. It appears from the studies mentioned that instructors may need to teach different limb-movement patterns depending on the task (ascending or descending) and ladder angle (vertical or angled). Two particular questions prompted the initiation of the study reported here. What are the different patterns of limb movement people use? How do these patterns differ in terms of safety?

### 1.3. Possible Limb-Movement Patterns

**Figure 1:**
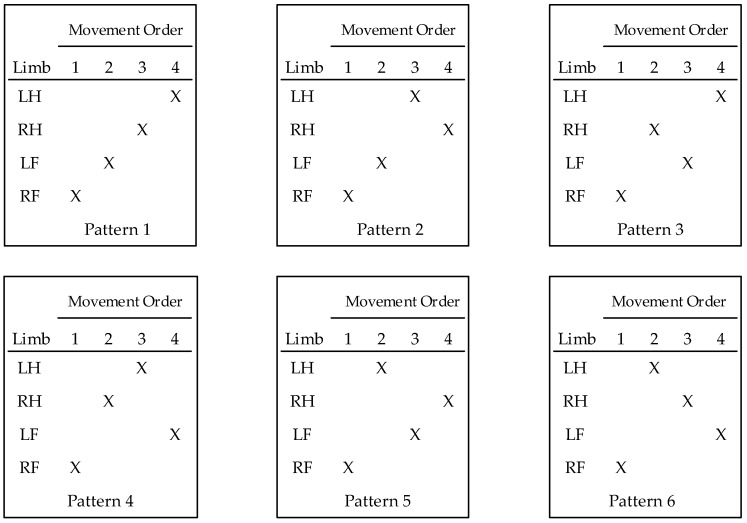
Hypothesized taxonomy of four-beat ladder climbing patterns with ordered limb movements. Limb notation: left hand (LH), right hand (RH), left foot (LF), right foot (RF).

The six limb-movement patterns in Figure 1 were identified as logically possible for four-beat movement patterns. Each pattern is based on an order in which one limb completes a movement before the following limb begins a movement. To explain the patterns, limbs are identified as right foot (RF), left foot (LF), right hand (RH), and left hand (LH). For consistency, each pattern is shown as starting with the RF moving off the floor to a rung. Each X indicates a movement of one limb, and the movement order proceeds left to right. To illustrate, the four-beat lateral pattern (RF RH LF LH) corresponds to Pattern 3 in Figure 1. The four-beat diagonal pattern (RF LH LF RH) corresponds to Pattern 5 in Figure 1. For descending, the patterns are reversed by finishing with a foot moving off one of the two lowest rungs toward the floor. Thus, for descent, the movement orders in Figure 1 are to be changed from 1, 2, 3, 4, to 4, 3, 2, 1.

Each of the cited studies contributes to the present state of knowledge, yet important questions have not been answered. The first concerns the taxonomy of limb-movement patterns depicted in Figure 1. Does the taxonomy include all patterns that might be used for three points of control climbing? Does the taxonomy include any patterns that are not practical or simply not used? All the cited studies [10,11,12,13,14,15,16,17,18] were based on defining the same four limb-movement patterns. In terms of the six possible patterns depicted in Figure 1, only Patterns 3 and 5 have been studied. Pliner and Beschorner mention they excluded from analyses subjects who had two successive hand movements per foot movement [11]. That would exclude Patterns 1, 2, 4, and 6 in Figure 1.

The first purpose of the present study was to determine if untrained students do in fact use all six patterns. A second purpose of this project was to explore a method for distinguishing the patterns in terms of relative safety. Both purposes were addressed by observing untrained students performing ladder climbing in a laboratory environment. The expectation was that findings would provide a foundation for two lines of future studies: (1) addressing patterns for using ladders with at least three points of control, and (2) seeking one or more best limb-movement patterns to use when teaching ladder climbing that uses three points of control.

## 2. Materials and Methods

### 2.1. Experimental Plan

The study was designed to obtain video observations of subjects performing four tasks: ascending an extension ladder (Task 1), descending the extension ladder (Task 2), ascending a vertical ladder (Task 3), and descending the vertical ladder (Task 4).

Due to concerns about the order of tasks having an influence, the starting task for subjects alternated between the two ascending tasks (Task 1 and Task 3). Their second task was descending the other ladder (Task 2 or Task 4).

Before starting the experiment, the experimental plan was approved by the applicable research ethics committee, the University of Montana Institutional Review Board (approval number 212-18). All subjects gave their informed consent for inclusion before they participated in the study.

### 2.2. Equipment

**Figure 2:**
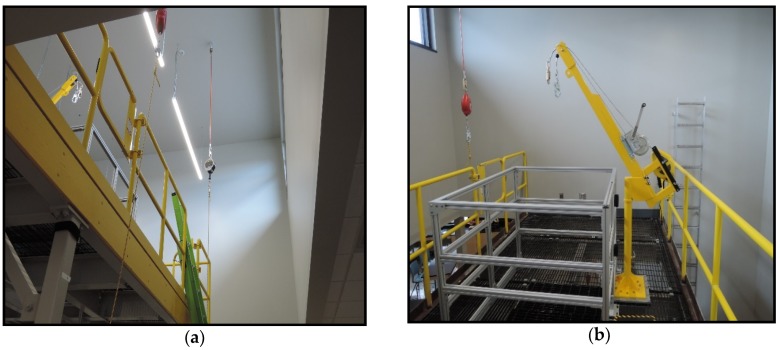
Personal fall arrest anchorages: (**a**) ceiling mounted anchor, (**b**) davit on the mezzanine.

The experiment used two ladders—an extension ladder and a fixed vertical ladder with rung spaced at 302 mm and 295 mm, respectively. Both ladders provided access to a mezzanine 3.93 m (10 Ft) high. The angle of the extension ladder was approximately 75° based on the Hepburn recommendation for optimal ladder stability for a ladder leaning against a wall [19]. The fixed ladder was straight vertical (90°) and built for this lab to access a hatch in the mezzanine. In order to ensure protection of subjects from falling, each was provided a fall-protection harness (DBI SALA Exofit brand). The harnesses came in sizes from extra-small to extra-large. Subjects were assisted with finding an appropriately sized harness and adjusting the straps. Before each task, subjects attached the D-ring in their harness to a retractable lifeline. The retractable lifelines were hanging from one of the anchors shown in Figure 2a for the extension ladder or Figure 2b for the fixed ladder. 

### 2.3. Experimental Procedures

Upon arriving at the lab, the participant’s attire was inspected to ensure compliance with the safety lab requirements—full length pants and closed toed shoes. After an oral explanation and time to review the consent form, all subjects indicated their consent by signing the consent form.

The tasks performed consisted of climbing and descending the extension ladder and the fixed ladder. Participants were instructed to climb either the fixed ladder or the extension ladder based on an alternating cycle. Cycle A starts with ascending the portable extension ladder. Cycle B starts with ascending the fixed ladder. Cycle A is used to explain the procedure. The participant would attach their harness D-ring to the retractable lifeline hanging from the ceiling-mounted anchor shown in Figure 2a. They would then climb the extension ladder and step onto the mezzanine. They would then disconnect the retractable lifeline, walk to the mezzanine hatch, hook their dorsal D-ring to the davit shown in Figure 2b, and proceed to descend the fixed ladder. After completing their descent and briefly pausing, they would ascend the fixed ladder. After stepping out of the hatch onto the mezzanine, they would disconnect from the davit, walk to the extension ladder, hook up to the ceiling anchor cable, descend, unhook, and doff their harness.

**Figure 3:**
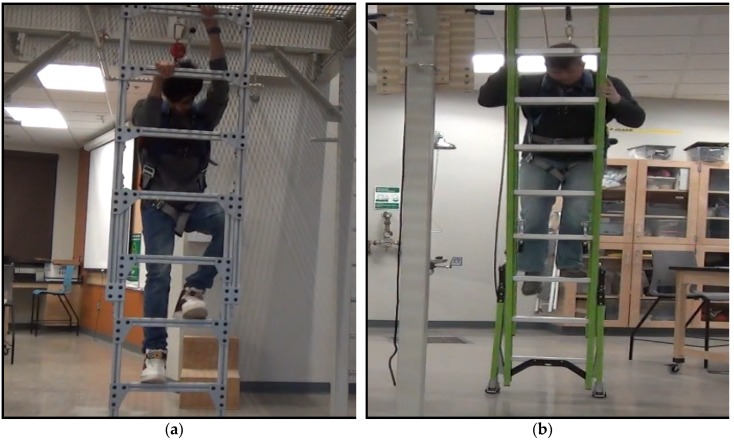
View of video camera: (**a**) fixed ladder climbing, (**b**) extension ladder descending.

The subjects were videotaped while ascending and descending the ladders. The video camera locations were chosen to obtain clear views of the fixed ladder pictured in Figure 3a and the extension ladder pictured in Figure 3b.

### 2.4. Participants

The subjects for this experiment were occupational safety and health students and civil engineering students. Requirements for participation were being over the age of 18 and being physically capable of ascending and descending ladders. The study plan was to recruit 20 subjects without consideration of gender. The resulting subjects consisted of 17 male and 3 female undergraduates.

### 2.5. Processing Videos

The videotape recordings were processed using a commercial program called Observer XT 11 [20]. From the video recordings, the portions selected for processing were the first five seconds of each ascent, and the last five seconds of each descent. For ascents, the timing started once the last foot came off the floor. For descent, the time interval would start five seconds prior to the last foot coming off a rung. Further processing of the 5-s observation periods used two methods. One was using a fixed-interval occurrence-sampling method involving pausing the video at half-second intervals in order to obtain snapshot-like images to determine foot and hand points of contact and points of control at that moment [21,22]. The second method was watching the limb movements between the half second intervals in order to determine the movement pattern.

The software had been programmed into premade analysis templates that facilitated observations of points of control and limbs in the process of moving. The template was coded so the investigator could enter for each limb if it was in control or not. For each foot, control was indicated by being on a rung. For each hand, control was indicated by being on a rung. The subjects who had their hand on a siderail or in the air were coded as not being in control. After entering each observation, the video played another half second. This was done for the first five seconds of the ascent and the last five seconds of the descent. Thus, the plan was to obtain 10 snapshot-like observations per task, with four tasks per subject, resulting in 40 snapshots per subject. However, in some snapshots the mezzanine blocked view of the subject’s hands so less than 10 observations were achieved.

The videos were also reviewed to determine the order of limb movement. When two limbs appeared to move simultaneously, the video was reviewed to determine which limb was first to contact the applicable rung. Using the limb movement order for each subject performing a task, the applicable pattern was determined.

The data were collected and organized into a large, raw data table using a Microsoft Excel^®^ spreadsheet. The table included for each snapshot (usually 10) the number of limbs in control, and for each task the limb-movement pattern.

### 2.6. Analyzing Data

For the first purpose, analysis consisted of determining from the video recordings the limb movement pattern used for each subject performing each task. The number of subjects using each pattern was subsequently tabulated.

For the second purpose, analyses started by deciding on a dependent variable to indicate the level of safe climbing practice. In an earlier study of ladder climbing, Hammer and Schmalz [18] compared five ladder inclinations in terms of the proportion of at least three limbs having contact. A similar measure was adopted for this study except points of control replaced points of contact. The difference is significant because Hammer and Schmalz counted hands grasping the siderails as a point of contact, whereas the present study does not. The variable indicating safe performance (*SP*) for this study was defined as the percentage of observations using three or four points of control.

$$SP=100\times \frac{\left(Number\;of\;3\;or\;4\;points\;of\;control\right)}{Total\;snapshots\;in\;the\;task}$$

Minitab 17 statistical software [23] was used to run Kruskal–Wallis (K-W) tests to compare *SP* for each of two independent variables: (1) the patterns, and (2) the four tasks.

Twenty subjects performed four tasks each for a total of 80 tasks performed. The plan was to obtain 10 snapshots of each to total 800. There were 13 missed due to being hidden from vision, making a total of 787 snapshots for analysis. These snapshots consisted of 200 siderail users and 587 rung users.

## 3. Results

### 3.1. The Taxonomy of Ladder Climbing Patterns

Observations of the video recordings of subjects performing 80 tasks permitted identifying a pattern in 79. One task could not be classified due to a mixing of the use of rungs and side rails. Out of the 79 tasks, 68 were classified as one of the six-patterns in the taxonomy developed prior to the study (Figure 1). The 11 unaccounted observations were subjects sliding their hands up or down the rails with no noticeable pattern. Figure 3b shows a subject using the side rails in a typical manner.

### 3.2. Points of Control by Pattern

Observations of the 587 snapshots of rung users were reviewed to determine the number of points of control. These involved 59 of the 80 tasks. Table 1 provides the number of tasks broken down by pattern. For each of these tasks, the percentages of one, two, three, and four points of control were determined. The snapshots showing three and four points of control were combined and used to compute the percentage within each task, i.e., the performance variable *SP*. Pattern p1 had the largest *SP* (76.7%). However, that does not mean p1 was statistically different from the other patterns. In order to examine possible statistical significance, the K-W test was used.

In order to properly use the K-W test, each factor should have at least five observations, and the observation should have a balanced distribution. In this case, the p1 and p4 patterns had less than five so they were excluded. The observations for the other four patterns were found to meet the requirement of balanced distribution.

The K-W test results are in Table 2. The median *SP* by pattern ranged from 60 to 75. The K-W test determines the pattern-specific mean rank by first putting all observations in rank order, then sorting the rank values back into their respective factor dataset. Using these rank values, the mean of each factor dataset was computed and shown in Table 2. The Z-value column indicates how the mean rank of each pattern compares to the mean rank of all observations. The test statistic (H) was computed from the mean rank values and compared to the chi-square distribution to determine the *p*-value (*p* = 0.14). The null hypothesis for the K-W test, that median ranks of the four factors do not differ in *SP*, led to the conclusion that the null hypothesis cannot be rejected with the observations from this experiment.

**Figure 4:**
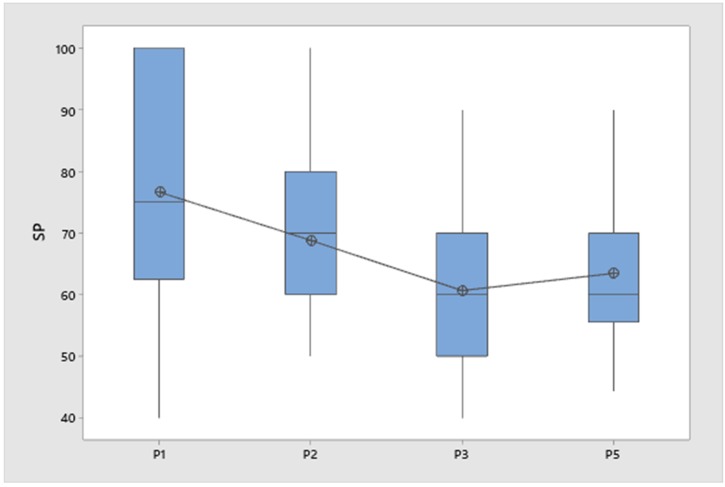
Box plot of four patterns showing *SP* means, medians, and interquartile ranges of observations.

Figure 4 provides a visual comparison of the factor-specific *SP* values used in the K-W test. It includes for each pattern the median, mean, and interquartile range.

### 3.3. Points of Control by Task

Out of 80 tasks performed by the 20 subjects, 59 showed subjects using the rung for their hands. Results of the experiment provided data suitable for examination if subjects used safer patterns for some tasks than others. Table 3 provides results of a K-W test for possible differences in the performance variable *SP*. The median value of *SP* ranged from 60 to 70. The mean ranks of the 59 observations ranged from a low of 26.4 for Task 1 to 35.3 for Task 2. These differences were not statistically significant (*p* = 0.51).

## 4. Discussion

The initial purpose of this projects was to determine if the hypothesized six-pattern taxonomy of different limb-movement patterns for ascending and descending ladders is complete. A second purpose was to compare the patterns in terms of safety. Both purposes were addressed by observing untrained students performing ladder climbing in a laboratory environment.

The use of undergraduate students as subjects was both a strength and a limitation. It was a strength in terms of learning about limb-movement patterns used by people with little or no training in safe ladder climbing. It was a limitation in terms of generalizability to the population of workers who use ladders in their work. Another limitation is the experiment used two ladders available in the laboratory. Therefore, influences of ladder features could not be explored.

### 4.1. Findings Regarding Hypothesized Taxonomy

All six hypothesized patterns were used by the subjects who used their hands to grip the rungs rather than the siderails. Patterns p2, p3, and p5 were used by most of these subjects. A seventh pattern was being used by subjects who were observed performing one or more of the tasks by sliding their hands on the siderails with no discernable gripping pattern.

A limitation of the study was that subjects performed each task only one time. It is possible that novice subjects performing the same tasks multiple times will change their limb-movement patterns as they gain confidence and skill.

### 4.2. Findings Comparing Patterns of Ladder Use

The second purpose of the project was to compare the patterns in terms of safety. The comparison was based on the variable *SP*, a performance variable defined as the percentage of snapshots using three or four points of control. The *SP* of the four patterns with sufficient data (p1, p2, p3, and p5) were not significantly different.

Multiple follow-on studies using larger sample sizes are needed to provide trainers with science-based information about the climbing patterns they teach. It would be useful to learn the safest pattern for each of the four tasks as well as to explore the possibility that optimal task-specific patterns may be related to the climber’s physical characteristics such as stature, limb length, or handedness. This would be invaluable for ladder-climbing instructors to justify teaching particular limb-movement patterns. A related line of study is recommended to explore the patterns in terms of time to develop proficiency.

## 5. Conclusions

In addition to confirming that all six hypothesized ladder-climbing patterns can be used, a second important conclusion comes from this project. That is the use of a measure of relative safety, *SP*, for comparing patterns of ascending and descending ladders. In this study, *SP* was based on the snapshot images obtained by using a fixed-interval sampling plan. However, the same measure of relative safety could be used for other sampling intervals, or for continuously obtained observations. The authors propose the *SP* can serve as a suitable performance variable for future research into limb-movement patterns while ascending and descending ladders.

## Figures and Tables

**Table 1:** Number of tasks classified by pattern and task-specific *SP* See article supplement Table S1 for details.

Pattern	Number of Tasks	*SP*
p1	6	76.7
p2	15	68.8
p3	15	60.7
p4	3	42.3
p5	16	63.5
p6	4	65.0
Total	59	

**Table 2:** Results of K-W test on patterns vs. use of three or four points of control.

Pattern	Observations	Median *SP*	Mean Rank	*Z*-Value ^1^
p1	6	75.0	36.1	1.65
p2	15	70.0	30.0	1.06
p3	15	60.0	21.3	−1.59
p5	16	60.0	24.5	−0.62
Overall	52		26.5	

^1^ H = 5.47, DF = 3, *p* = 0.141 (adjusted for ties).

**Table 3:** Results of K-W test on tasks vs. use of three or four points of control. See article supplement, Table S2 for details.

Task	N Tasks	Median *SP*	Mean Rank	*Z*-Value ^1^
1	13	60.0	26.4	−0.86
2	11	70.0	35.3	1.13
3	17	60.0	27.4	−0.74
4	18	65.0	31.9	0.55
Overall	59		30.0	

^1^ H = 2.31, DF = 3, *p* = 0.510 (adjusted for ties).

## Supplementary Materials

The following are available online at https://www.mdpi.com/1660-4601/17/8/2897/s1, Table S1: Observed Percentage of Three and Four Points of Control, Ordered by Pattern, Table S2: Observed Percentage of Three and Four Points of Control by Task.
