# Supplementary material for: Ladder Safety: A Taxonomy of Limb-Movement Patterns for Three Points of Control
Source: Int J Environ Res Public Health. 2020 Apr 22;17(8):2897. doi: 10.3390/ijerph17082897 (PMC7215462; doi:10.3390/ijerph17082897)
Supplement: Supplementary file 1 [file ijerph-17-02897-s001.pdf]

International Journal of
Environmental Research and Public Health

MDPI

Supplementary Data

Article Title

Ladder safety: A taxonomy of limb-movement patterns for three-points of control

Table S 1. Data Used for Table 1: Observed Percentage of Three and Four Points of Control, Ordered by Pattern

Subject	Task	Obs%	Pattern
18	2	70	1
17	3	100	1
11	1	70	1
10	3	40	1
9	2	80	1
2	3	100	1
19	4	80	2
18	3	80	2
17	4	100	2
16	3	50	2
15	2	70	2
13	3	50	2
12	1	70	2
12	2	60	2
12	4	70	2
10	4	60	2
9	1	62.5	2
5	4	70	2
4	1	60	2
3	4	60	2
2	4	90	2
20	1	40	3
20	2	50	3
17	1	80	3
17	2	90	3
14	2	70	3
14	3	70	3
14	4	60	3
10	1	50	3
10	2	50	3
9	3	50	3
7	4	50	3
5	1	60	3
5	2	70	3
4	4	60	3
3	1	60	3

8	1	60	4
6	3	40	4
5	3	57	4
20	3	55.5	5
20	4	44.4	5
19	3	70	5
18	4	80	5
16	4	70	5
14	1	60	5
13	4	60	5
11	3	50	5
11	4	50	5
9	4	55.5	5
8	3	70	5
8	4	70	5
7	2	90	5
6	2	60	5
6	4	70	5
3	3	60	5
12	3	70	6
7	1	70	6
7	3	70	6
6	1	50	6
Sum: 59			59

Table S 2. Data used for Table 3: Observed Percentage of Three and Four Points of Control by Task

	Task 1	Task 2	Task 3	Task 4
	60	70	100	90
	60	60	60	60
	60	90	57	60
	50	80	40	70
	70	50	70	70
	60	60	70	50
	62.5	70	50	70
	50	70	40	55.5
	70	90	50	60
	70	70	70	50
	60	50	50	70
	80		70	60
	40		50	60
			100	70
			80	100
			70	80
			55.5	80
				44.4
N	13	11	17	18
Median SP	60	70	60	65
